# Egg Predation by the Introduced Lady Beetle, *Coccinella septempunctata* (Coleoptera: Coccinellidae), Lowers Mortality but Raises Relative Risk for the Native Lady Beetle, *Coccinella novemnotata*

**DOI:** 10.1371/journal.pone.0118493

**Published:** 2015-06-19

**Authors:** Rakim Turnipseed, Todd A. Ugine, John E. Losey

**Affiliations:** 1 Department of Environmental Science, Policy, & Management, University of California, Berkeley, CA, 94720, United States of America; 2 Department of Entomology, Cornell University, Ithaca, NY, 14853, United States of America; Swedish University of Agricultural Sciences, SWEDEN

## Abstract

Populations of the native ninespotted lady beetle, *Coccinella novemnotata* Herbst, have undergone precipitous declines in North America following the establishment of the exotic sevenspotted lady beetle, *Coccinella septempunctata* L. Recent volunteer efforts have made it possible to establish colonies of the now-rare *C*. *novemnotata* and test mechanisms contributing to its decline. We evaluated the relative frequencies of intraguild predation and cannibalism of eggs between these two species. A single *C*. *novemnotata* or *C*. *septempunctata* adult was exposed to conspecific and heterospecific eggs in either the presence or absence of pea aphids. The study revealed two expected results: 1) eggs of *C*. *novemnotata* were consumed more frequently than eggs of *C*. *septempunctata* by both species, and 2) egg consumption was higher when aphids were absent, independent of predator and egg species. There were also two unexpected results from the study: 1) the asymmetry between egg predation rates was higher when aphids were present, and 2) higher predation rates on *C*. *novemnotata* eggs in the absence of alternate prey was due to a relatively higher rate of intraspecific cannibalism. This implies that *C*. *novemnotata* would have suffered higher egg mortality rates before the invasion of *C*. *septempunctata*, but even though the aggregate rate of egg predation on *C*. *novemnotata* eggs is lower post-invasion, it is still significantly higher than the aggregate rate of predation of *C*. *septempunctata* eggs. This differential pattern of asymmetric predation could contribute to habitat compression and the overall decline of *C*. *novemnotata*.

## Introduction

Before the mid 1980s, *Coccinella novemnotata* Herbst was the most commonly encountered native lady beetle species in the northeastern region of the United States and among the most common nationally [1]. Since then, *C*. *novemnotata* and other native coccinellids, such as *Coccinella transversoguttata richardsoni* Brown and *Adalia bipunctata* L., have undergone widespread population declines throughout North America [1, 2] following the introduction of exotic coccinellids for pest control, including *Coccinella septempunctata* L. [3]. Because of the close timing between the extirpation of native and establishment of exotic coccinellids, it has been suggested that *C*. *septempunctata* may have contributed to the decline of native coccinellids, including *C*. *novemnotata* [4, 5, 6, 7, 3, 8]. Among several proposed mechanisms behind the widespread decline of native coccinellids are competition with and intraguild predation by invasive coccinellids [9, 10, 11, 12, 13].

Several invasive coccinellid species, such as *C*. *septempunctata* and *Harmonia axyridis* Pallas, are known to be intraguild predators of native North American coccinellids when aphid populations become scarce [14, 15, 16, 17, 18, 6, 19]. Additionally, it has been shown that coccinellid eggs are particularly vulnerable to intraguild predation and that they can provide a better source of nutrition to the consumer than aphids in terms of larval development [20, 13]. For example, it has been demonstrated in a laboratory study [20] that *H*. *axyridis* larvae can complete development on a diet of the eggs of two native coccinellid species, *Coleomegilla maculata* De Geer and *Olla v-nigrum* Mulsant, however the same two native species were not able to complete development when provided only *H*. *axyridis* eggs. The presence of alternative prey has been shown to impact predation rates among coccinellids and other animals [21, 22, 23, 24, 25]. For example, it has been demonstrated that aphid density and egg consumption by fourth-instar coccinellid larvae were inversely proportional [14].

Not all egg predation is due to intraguild interspecific predation; cannibalism is also a well-documented phenomenon within Coccinellidae. *C*. *septempunctata* have been reported to cannibalize their own eggs [26], and that they prefer to eat their own eggs versus those of *A*. *bipunctata*. The introduced species *H*. *axyridis* have been reported to cannibalized their own eggs [1], although they showed no preference for conspecific versus heterospecific eggs. Adults of the native species *Cycloneda sanguinea*, have been shown to cannibalize their own eggs, and did so more compared to heterospecific eggs [27]. It has been suggested that when some coccinellid species cannibalize, they obtain a higher quality diet from their conspecific prey, which results in higher fitness [28]. They showed that *H*. *axyridis* achieved higher rates of survival and a reduced time to adult eclosion on a diet of conspecific prey than aphids of poor quality and hypothesize that this could be a function of their ability to detoxify their prey more readily as compared to heterospecific prey. In another study, [29] demonstrated that egg cannibalism by the larva of a coccinellid species, *Hippodamia variegata* Goeze, resulted in increased fitness. However, cannibalism is not always advantageous to the cannibal in fitness terms. It has been demonstrated that cannibalism reduces juvenile survival significantly in some coccinellids [30]. In one study, it was demonstrated that fourth instar larvae of *C*. *septempunctata* took longer to develop on a diet of conspecific eggs compared to an aphid-based diet [26].

While there is great deal of literature on coccinellid egg predation originating from laboratory studies, there is relatively little stemming from field studies. In one of the notable exceptions [31], it was reported that over 40% of sentinel eggs of the native species *Col*. *maculata* were attacked within 48 hours of being placed in the field and that the coccinellid complex in the fields where the experiment took place were dominated by exotic species. In another study that also used sentinel coccinellid eggs [32], it was determined that eggs of native species suffered significantly higher rates of predation than those of exotic species, and that exotic ccoccinellid species were more abundant as in the previous study. Although these results suggest that intraguild predation by exotic species on native coccinellid eggs was occurring, direct observation of egg predation revealed very few incidences involving exotic coccinellids and most of the observed predation events involved non-coccinellid predators [32].

While this combination of laboratory and field studies has started to provide a framework for evaluating the impact of exotic conccinellids on native species in North America through the mechanism of egg predation, no study to date has examined an exotic/native pair from the same genus. This represents a conspicuous absence of data since congeneric species might be predicted to have a high degree of niche overlap. The native *C*. *novemnotata* and the exotic *C*. *septempunctata* clearly fall into this category, but egg predation or other modalities of interaction have heretofore been impossible to examine because *C*. *novemnotata* had become so rare that individuals were not available for research. Fortunately, volunteers working with the Lost Ladybug Project (www.lostladybug.org) have recently discovered several populations of *C*. *novemnotata* and individuals have been collected and used to establish research colonies.

Rearing constraints (e.g. cost and timing) and concerns for the potential spread of disease [33, 34] or suboptimal genetic combinations [35] associated with high density laboratory populations precluded field studies with *C*. *novemnotata*, so we implemented a laboratory study (with inherently lower risk of beetle escape) to gauge the levels of egg predation within and between *C*. *novemnotata* and *C*. *septempunctata* in the presence and absence of aphid prey.

## Materials and Methods

Our lady beetle colonies were initiated with adults collected from southeastern Oregon, southwestern South Dakota, and Long Island New York in 2012. Eggs of *C*. *novemnotata* and *C*. *septempunctata* were obtained from these laboratory colonies. Colony beetles were reared singly in 44mL plastic cups containing a single 2x7cm strip of dry paper towel, and were provided an ad libitum diet of pea aphids, *Acyrthosiphon pisum* Harris (Hemiptera: Aphididae), that were reared on fava bean plants, *Vicia faba* L. (Fabales: Fabaceae). All insect colonies were maintained at 25±2°C and a 16:8h light:dark cycle in an environmental incubator.

We conducted a 2x2x2 factorial study investigating the effect of predator species (*C*. *novemnotata* and *C*. *septempunctata*), egg species (*C*. *novemnotata* and *C*. *septempunctata*), and aphid density (presence or absence) on egg consumption. Newly-eclosed adult *C*. *novemnotata* and *C*. *septempunctata* were held for 24h without food prior to determining their sex. Because adult males might not spend as much time as females in oviposition sites, and because they have been reported to have statistically indistinguishable egg predation rates compared to females [14] only adult female beetles were used in trials. This also served to minimize the variation among treatments. Adult females were introduced singly into 20ml plastic cups that contained a group of three conspecific eggs (<24h old) and a group of three heterospecific eggs (<24h old). The eggs were placed on opposite sides of the cup approximately 2 cm apart. Each cup received either 20 third-to-fourth instar aphids (0.035±0.001 grams) or no aphids. The selected density of approximately 20 aphids was chosen because it has been shown to be optimal for fitness of *C*. *novemnotata* (Losey et al. 2012), and we assume it was a reasonable amount of aphids for *C*. *septempunctata* as it is of similar size and occupies a similar ecological niche. Each cup was observed for an hour or until all eggs in the cup had been consumed. For each cup we recorded the predator species, presence or absence of aphids, and the number of eggs remaining for each species of egg. The experiment was conducted across three dates (completely randomized block design with dates as blocks) and a total of 40 replicate cups containing *C*. *novemnotata* as predator (n_eggs_ = 240) and 40 replicate cups containing *C*. *septempunctata* as predator (n_eggs_ = 240) were monitored. We also measured the length and width of three arbitrarily selected eggs from each of five female beetles of each species to test for difference in egg volume, which could play a role in egg appearance and subsequent consumption by adult lady beetles.

### Statistical Analyses

All statistical analyses were performed with the statistical package JMP Pro version 9 [36]. Total egg consumption across eggs species was analyzed using analysis of variance (ANOVA). Our model included the fixed effects predator species, aphid density and the two-way interaction (e.g. 2x2 factorial analysis). Because adult lady beetles had a choice of eggs to eat, we also analyzed the difference between the number of *C*. *novemnotata* versus *C*. *septempunctata* eggs consumed as a function of the fixed effects predator species, aphid presence/absence, and the predator species by aphid presence/absence interaction. This approach takes into account the paired nature of the data and allowed us to investigate the effect of egg species, making the final model a 2x2x2 factorial. Post-hoc analyses were conducted using Tukey’s HSD at alpha = 0.05. Egg volume was analyzed using ANOVA and our model included the fixed effect lady beetle species. We also blocked our measurements by the female that laid the eggs.

Using the egg predation data generated in our experiment, we modeled egg predation across historical time periods pertinent to *C*. *novemnotata’s* decline. We defined predation regimes based on the presence and relative density of these two species as they would have been during “pre-invasion”, when all predators would have been *C*. *novemnotata*, versus “post-invasion”, when the two species would have interacted. Our post-invasion complex was defined as equal densities of *C*. *septempunctata* and *C*. *novemnotata*. Since *C*. *septempunctata* was not present pre-invasion, we used the 20 *C*. *novemnotata* replications as a measure of pre-invasion egg predation rates. To construct a comparable data set for the post-invasion era we randomly selected 10 values from the 20 *C*. *novemnotata* replications and concatenated 10 randomly selected values from the 20 *C*. *septempunctata* replications. The size of these generated sets was capped at 20 observations to maintain proportional variance with the measure data sets of 20 observations each. The result for each iteration was a set of 20 values equally distributed between *C*. *novemnotata* and *C*. *septempunctata* and the process was repeated 1500 times to generate a mean and standard error. We used this algorithm to generate post-invasion distributions for both *C*. *novemnotata* eggs eaten and *C*. *septempunctata* eggs eaten. We tested the difference between pre- and post-invasion predation rates on *C*. *novemnotata* eggs by evaluating the number of iterations of post-invasion data for which the mean equaled or exceeded the mean pre-invasion value for *C*. *novemnotata*. Specifically, for this test p = ∑(mean post invasion ≥ mean pre-invasion)/total iterations. We used the same algorithm to test the difference between the mean post-invasion rates for *C*. *novemnotata* and *C*. *septempunctata* using the generated mean for *C*. *novemnotata* as the fixed comparison value. All calculations for the resampling analyses were implemented in Microsoft Excel.

## Results

**Fig 1:**
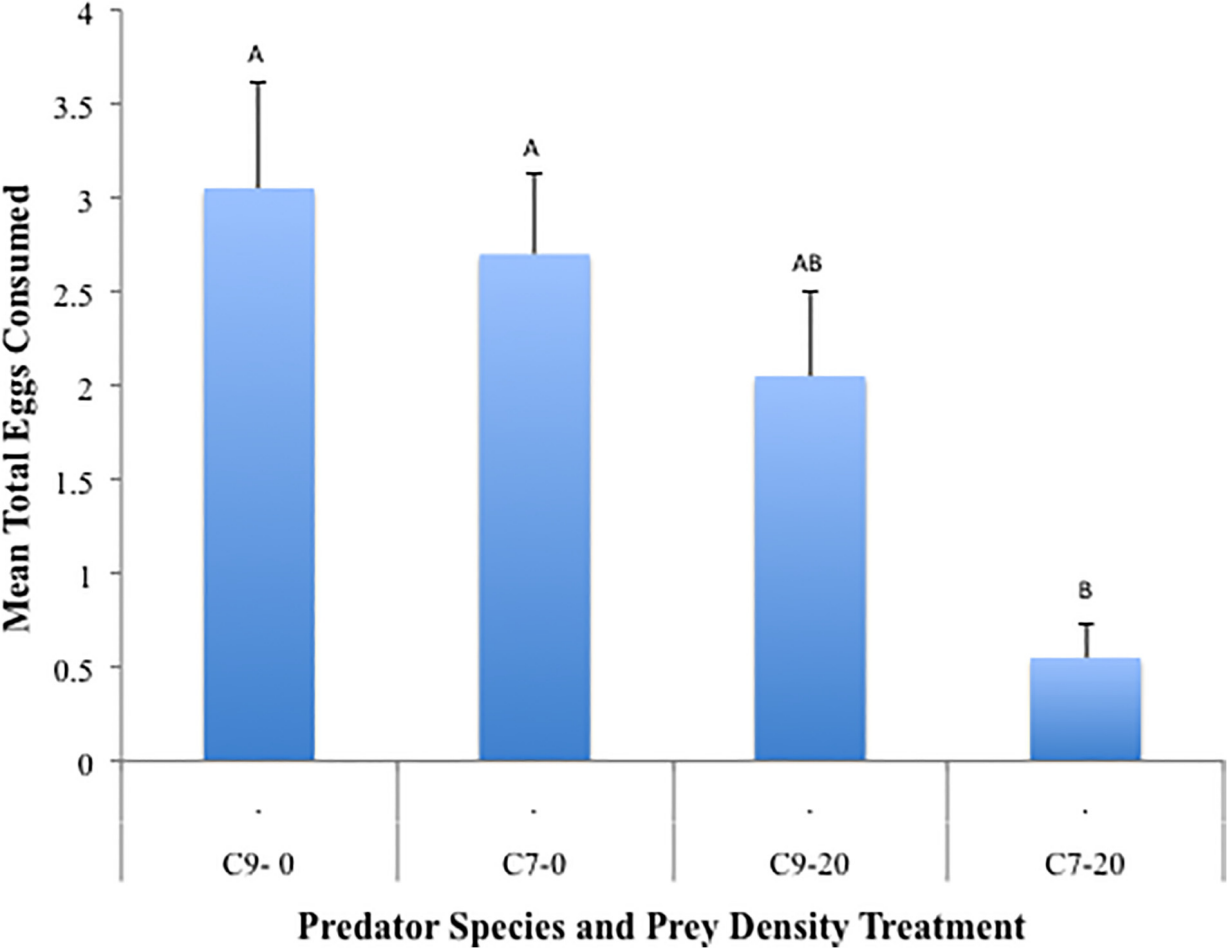
Mean total eggs consumed. Each bar represents mean (+SEM) egg consumption per predator for a single treatment combination across two factors, predator (C9 = *C*. *novemnotata*, C7 = *C*. *septempunctata*), and aphid density (0 or 20 pea aphids). Columns with the same letter are not significantly different (*p* > 0.05).

The mean total number of eggs consumed was significantly affected by the main effect of predator species (*p* = 0.03, Fig 1); *C*. *novemnotata* consumed more eggs (mean ± SE: 2.55 ± 0.37) than *C*. *septempunctata* (1.63 ± 0.29). There was also a significant effect of aphid density on the number of eggs consumed (*p* = 0.0004). In the absence of aphids, an average of 2.88 ± 0.35 eggs were consumed versus only 1.30 ± 0.27 eggs when 20 aphids were present. There was not a significant predatory species by aphid density interaction (*p* = 0.18).

**Fig 2:**
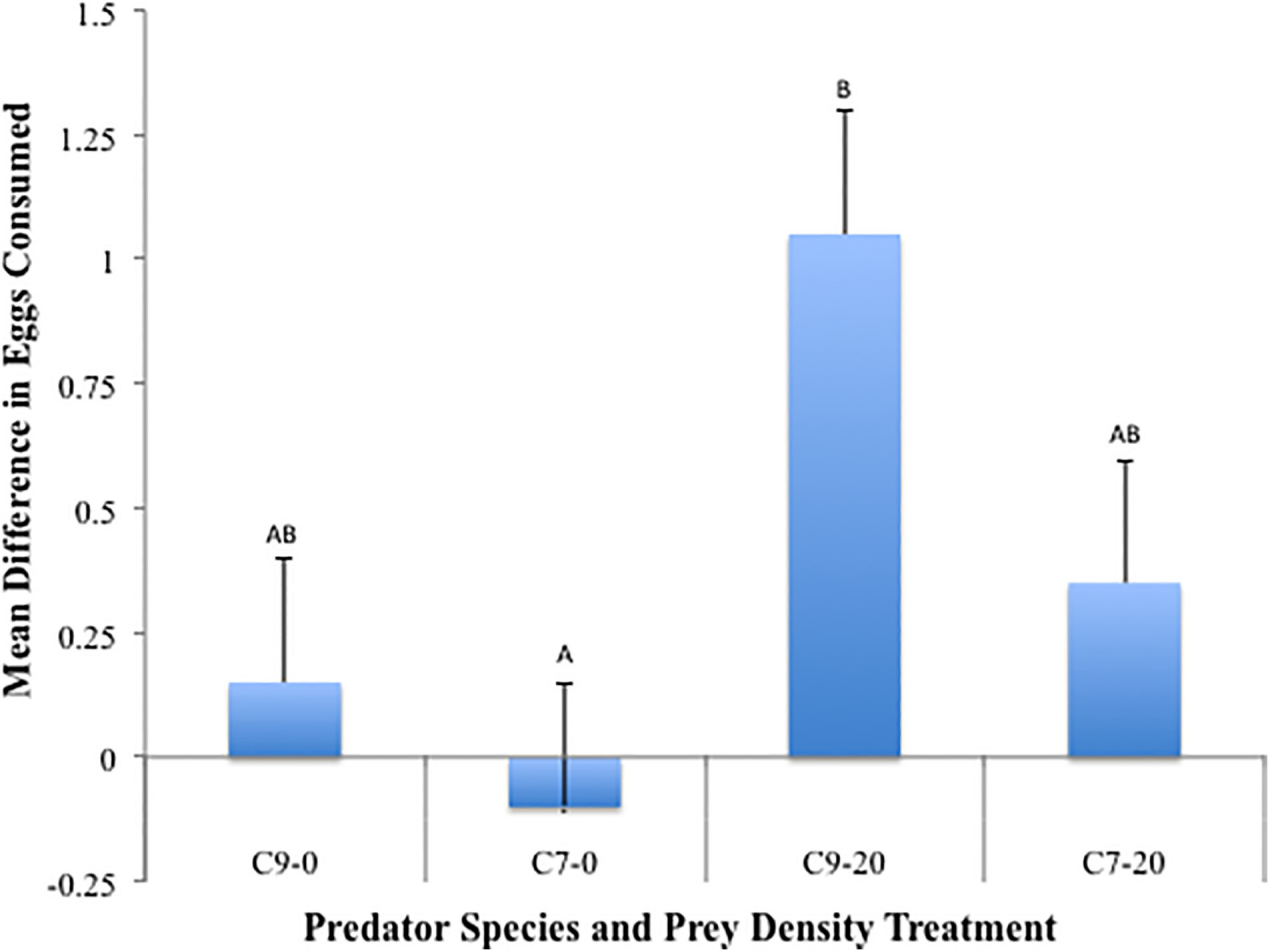
Mean difference in egg consumption. Each bar represents mean (+SEM) difference in consumption per predator (*C*. *novemnotata* eggs—*C*. *septempunctata* eggs) for a single treatment combination across two factors, predator (C9 = *C*. *novemnotata*, C7 = *C*. *septempunctata*), and aphid density (0 or 20 pea aphids). A value of 0 represents no difference in consumption of the eggs of the two species. Columns with the same letter are not significantly different (*p* > 0.05).

Across predator species and aphid densities an average of 0.36 (12%) more *C*. *novemnotata* eggs were consumed versus *C*. *septempunctata* eggs (*p* = 0.02). The difference in egg consumption across egg species was not significantly higher when *C*. *novemnotata* was the predator (0.60 ± 0.19) compared to the difference when *C*. *septempunctata* was the predator (0.13 ± 0.23; *p* = 0.11). Aphid density had a significant affect on the differential consumption of egg species (*p* = 0.02). The asymmetry in consumption between egg species was positive across both aphid densities indicating more *C*. *novemnotata* eggs were consumed than *C*. *septempunctata* eggs. The difference in egg consumption was higher when aphids were present (0.70 ± 0.18) compared to when aphids were absent (0.03 ± 0.23). Across predator species and aphid densities (Fig 2) all differences were relatively close to 0 indicating nearly random consumption of the two egg species and there was not a significant interaction between aphid density and predator species (*p* = 0.44). The only significant departure from random consumption occurred when *C*. *novemnotata* was the predator and aphids were present. For that treatment combination, significantly more *C*. *novemnotata* eggs were consumed (*p* = 0.002). There was not a significant difference in the size (volume) of lady beetle eggs as a function of lady beetle species (*p* = 0.25).

**Fig 3:**
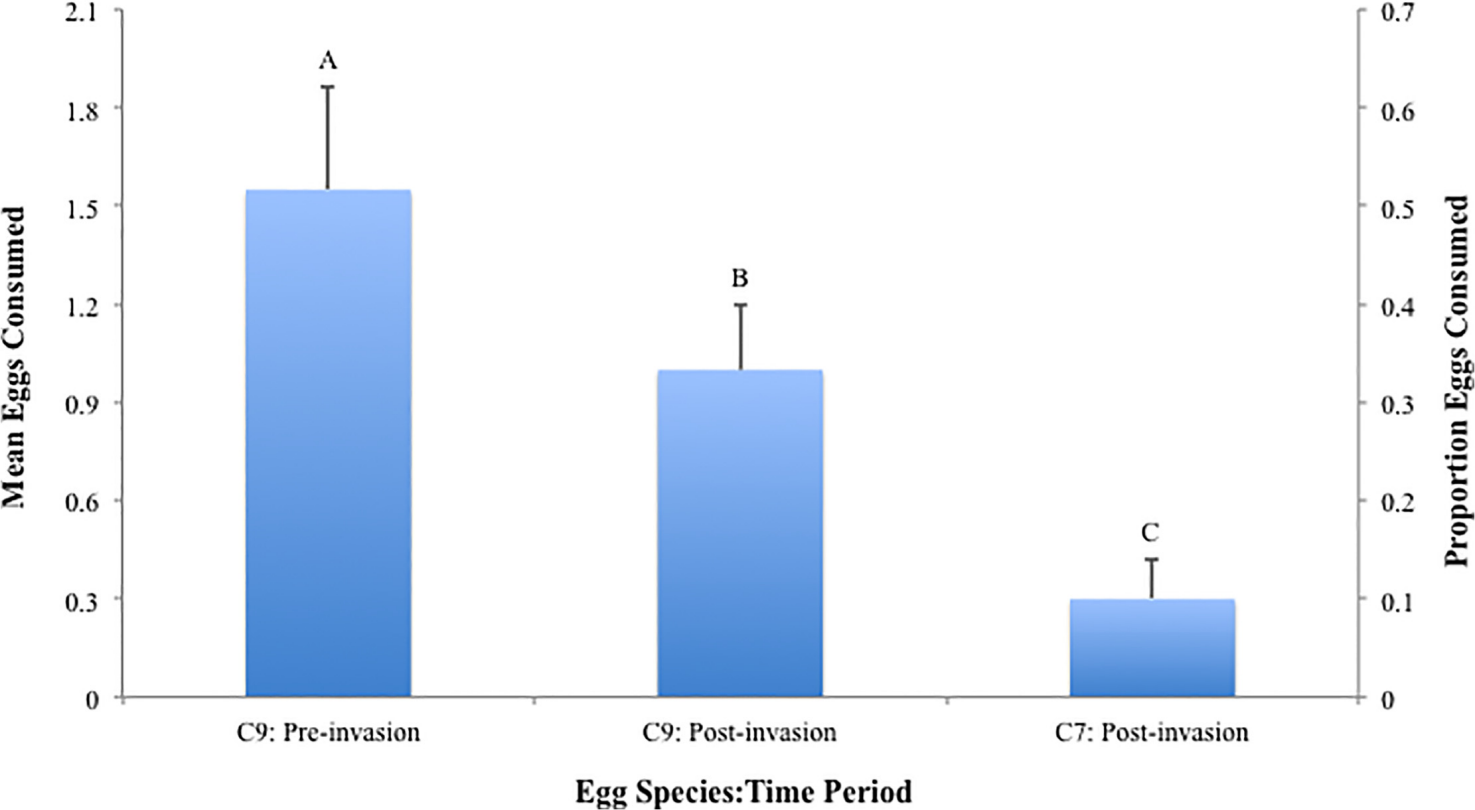
Mean egg consumption pre and post invasion. Each bar represents mean (+SEM) egg consumption per predator for a single treatment combination across two factors, predator species (C9 = *C*. *novemnotata*, C7 = *C*. *septempunctata*), and time period (pre-invasion or post-invasion). The invader species, *C*. *septempunctata*, was not present pre-invasion. Columns with the same letter are not significantly different (*p* > 0.05).

Across time periods, the mean number of *C*. *novemnotata* eggs consumed in the pre-invasion era was significantly higher than the number consumed in the post-invasion era (*p* = 0.013; Fig 3). The post invasion predation rate on *C*. *septempunctata* eggs was significantly lower than the post invasion rate on *C*. *novemnotata* eggs (*p* < 0.0001).

## Discussion

The result of this study with the most direct implications for the interaction between *C*. *novemnotata* and *C*. *septempunctata* was that *C*. *novemnotata* eggs were more likely to be consumed across predator species and aphid densities. The asymmetry in predation rates corroborates previous laboratory [20] and field studies [31, 32] that found higher rates of predation on the eggs of native species. The relative advantage conferred to *C*. *septempunctata* by the lower rates of egg predation represents a mechanism through which *C*. *novemnotata* may have been displaced throughout North America and adds to recent reports of other mechanisms including greater competitive ability in the larval stage [19] and a higher intrinsic rate of population increase [37].

While the asymmetry of egg predation suffered by these two species followed the direction expected based on previous studies, the specific pattern of egg predation by the two species across aphid densities was unexpected. More eggs were consumed when the aphids were absent [38, 39], but the asymmetry between predation on *C*. *novemnotata* eggs and *C*. *septempunctata* eggs was highest when aphids were present. This implies that future studies should be designed and previous studies perhaps reinterpreted based on the availability of alternate prey.

Even more surprising was the finding that when alternative prey were present it was not intraguild predation by *C*. *septempunctata* that had the greatest impact on *C*. *novemnotata* eggs, but rather intraspecific cannibalism by *C*. *novemnotata*. It appears that what gives *C*. *septempunctata* an advantage is not its voracity in eating a competitor’s eggs, but the behavioral flexibility to decrease cannibalism when other resources are available. Cannibalizing eggs or even an individual’s own offspring is not necessarily maladaptive. Consumption of “trophic eggs” can increase fitness by recycling scarce resources and coccinellids have recently been the first non-eusocial insect taxon that demonstrates trophic egg plasticity in response to varying resources in the environment [40]. Other studies have demonstrated that intraguild predation and cannibalism between larval coccinellids may result in nutritive costs that positively or negatively affect development [20, 13, 28, 29]. The mechanism for distinguishing between eggs would appear to be based on some factor other than size as our measurements revealed no significant difference in egg volume between the two species. This follows earlier reports that egg size was not a function of body size [41] and implies that there may be differences in chemical composition of the eggs. We did not measure any fitness parameters of the individual beetles we tested so we cannot determine if egg consumption affected fitness. However, our results raise the interesting possibility that *C*. *novemnotata* ‘s propensity for cannibalism was adaptive for pre-invasion conditions and its inability to shift that propensity when it rapidly came into contact with *C*. *septempunctata* (ostensibly a close competitor) contributed to its decline.

This predation pattern leads to an even more unexpected, even counter-intuitive, result. Considering only the conditions where aphid were present (which is where these density-dependent predators would most likely be), adding *C*. *septempunctata* with its lower egg predation rate to the system may have driven the mortality rate for *C*. *novemnotata* eggs lower in the post-invasion era than it had been in the pre-invasion era. Even though the addition of *C*. *septempunctata* may have lowered the rate of mortality for *C*. *novemnotata* in the egg stage, the high rate of cannibalism by *C*. *novemnotata* would still leave it at a disadvantage relative to *C*. *septempunctata*, which exhibits a very low propensity for cannibalism. Both the inability to rapidly evolve optimal new behavioral regimes and the specific relationship of intraguild predation and cannibalism could have important implications for other systems.

We acknowledge that confining beetles to containers under experimental conditions is not necessarily reflective of their behavior in natural environments where they have much more freedom to find additional alternative prey or avoid aphid or egg prey. However, complex interactions such as intraguild predation are likely to occur in aphidophagous guilds between different coccinellid species [9, 23] given that multiple aphidophagous insect species should be attracted to sites where aphid populations are high [42].Especially for native species that have become so rare do to their interactions with invasive species that field studies are no longer possible, the only alternative may be to discern the mechanism of past interactions or even predict future trends by conducting more controlled behavioral and or physiological studies.
